# Endothelium-Independent Vasorelaxant Effect of *Ligusticum jeholense* Root and Rhizoma on Rat Thoracic Aorta

**DOI:** 10.3390/molecules200610721

**Published:** 2015-06-10

**Authors:** Bumjung Kim, Kyungjin Lee, Khanita Suman Chinannai, Inhye Ham, Youngmin Bu, Hocheol Kim, Ho-Young Choi

**Affiliations:** Department of Herbology, College of Korean Medicine, Kyung Hee University, Seoul 130-701, Korea; E-Mails: ori-pharm@hanmail.net (B.K.); dostudy@naver.com (K.L.); sumankhanita6@gmail.com (K.S.C.); iham@khu.ac.kr (I.H.); ymbu@khu.ac.kr (Y.B.); hckim@khu.ac.kr (H.K.)

**Keywords:** *Ligusticum jeholense*, vasorelaxant effect, potassium channels, calcium channels, hypertension

## Abstract

*Ligusticum jeholense* has been used as the traditional medicine ‘Go-Bon’ (Chinese name, Gao-ben) in China and Korea. Considering the increased use of medicinal herbs to treat hypertension, in this study, we aimed to investigate the mechanisms of the vasorelaxation effect caused by *L. jeholense*. We tested the methanol (MeOH) extract of *L. jeholense* root and rhizoma for vasorelaxant effects; while using an isolated organ-chamber technique, *L. jeholense* extract (LJE) induced relaxation in the rat aortic rings by stimulating vascular endothelial and smooth muscle cells. LJE showed concentration-dependent relaxant effects on endothelium-intact and endothelium-denuded aortic rings pre-contracted with both phenylephrine (PE) and potassium chloride (KCl) in Krebs-Henseleit (KH) buffer. The vasorelaxant effect of LJE was partly attenuated by pre-treatment with glibenclamide or 4-aminopyridine (4-AP) as K^+^ channel blockers. Moreover, LJE showed concentration-dependent inhibition of vasoconstriction by Ca^2+^ supplementation in the aortic rings that were pre-contracted with PE or KCl in Ca^2+^-free KH buffer. In addition, a combination of LJE and nifedipine, pre-incubated further, decreased PE-induced contractions. The results suggested that LJE-induced vasorelaxation were related to blocking K^+^ channels and inhibiting entry of extracellular Ca^2+^ via receptor-operative Ca^2+^ channels (ROCCs) or voltage-dependent Ca^2+^ channels (VDCCs).

## 1. Introduction

High blood pressure, or hypertension, is a major determinant of mortality caused by cardiovascular disease, cerebrovascular disease, and stroke. In hypertension, vasoreactivity is an inevitable factor for the treatment of hypertension because it affects circulation and blood pressure in the cardiovascular system. Quality of life is important for antihypertensive therapy. However, although these drugs continue to be developed to treat hypertension, synthetic drugs have various adverse effects. The efficacy of these drugs increases in a dose-dependent manner, which leads to more adverse effects [1]. In addition to synthetic drugs, the use of herbs or herbal extracts is increasing in China, Japan, and Korea [2]. Recently, the use of natural herbs has shown a steady growth because of low toxicity and well-established therapy [3]. Many plants used in traditional medicine have been investigated for treating cardiovascular disease [4].

The genus *Ligusticum* (Umbelliferae), consisting of approximately 50 species growing in Asia, Europe, and North America, is complex and widespread [5]. The root and rhizoma of *Ligusticum* species are generally used in traditional medicine in China, Japan, and Korea to treat headaches, arthralgia [6], pain, common cold [7], and cardiovascular disease such as angina pectoris [8]. Recent studies have shown that these medicinal plants have anti-infective, sedative, analgesic [9], anti-cancer [10], anti-inflammatory [11], anti-mycobacterial [12], anti-nociceptive activity [13], anti-neuroinflammatory [14], and vasorelaxation effect [8].

*Ligusticum jeholense* Nakai et Kitagawa has been used as the traditional medicine, ‘Go-Bon,’ (Chinese name, Gao-ben) in China and Korea. Seven compounds in the root and rhizoma of *L. jeholense* were identified, such as levistolide A., xiongterpene, linoleic acid, sucrose, daucosterol, ferulic acid, and beta-sitosterol [15]. The main components from the *L. jeholense* essential oil were isolated, such as *m*-diaminobenzene, ligustilide [7], and β-phellandrene [5].

There are only a few pharmacological studies about this medicine, describing its anti-bacterial and anti-oxidant activity [7]. Although vascular activity was previously reported for the essential oil of *L. jeholense* [16], no study has investigated the vasorelaxation effects caused by *L. jeholense*.

Thus, considering the increased use of medicinal herbs to treat hypertension, in this study, we aimed to investigate the mechanisms of the vasorelaxation effect caused by *L. jeholense*. We found that the methanol (MeOH) extract of *L. jeholense* root and rhizoma (LJE) has vasorelaxant effects; while using an isolated organ-chamber technique, LJE induced relaxation in the rat aortic rings by stimulating vascular endothelial and smooth muscle cells. To our knowledge, this is the first report to demonstrate the vasorelaxation mechanisms of *L. jeholense* in Korea.

## 2. Results and Discussion

### 2.1. Effect of LJE on Phenylephrine (PE)- or Potassium Chloride (KCl)-Induced Contraction

We investigated the concentration-dependent vasorelaxant effects of LJE (30–500 μg/mL) on endothelium-intact aortic rings pre-contracted with PE (1 μM) or KCl (60 mM) in standard Krebs-Henseleit (KH) buffer. The vasorelaxant effect of LJE was calculated as a percentage of the relaxation in response to PE and KCl on the aortic rings.

**Figure 1:**
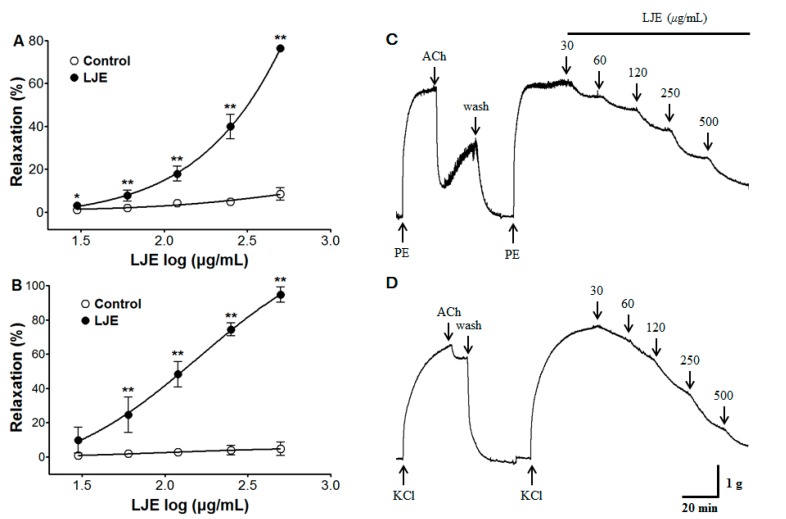
The concentration-dependent relaxation effect of LJE (30–500 μg/mL) in the presence or absence (control) of LJE on PE (1 μM) (**A**) or KCl (60 mM) (**B**) pre-contracted aortic rings. The traces of LJE induced-relaxant in endothelium-intact aortic rings pre-contraction by PE (**C**) or KCl (**D**). Values are expressed as mean ± SEM (*n* = 4–7). * *p* < 0.05, ** *p* < 0.01 *vs.* control.

LJE caused concentration-dependent relaxation in endothelium-intact aortic rings pre-contracted by PE or KCl treatment. The maximal relaxant effect was 76.4 ± 1.2% and 94.6 ± 4.5% at the concentration of LJE (500 μg/mL), respectively (Figure 1). In this study, we found the optimal concentration to generate a complete dose-response for LJE by studying the results of several tests. We applied the same concentration and equilibration time throughout the experimental period.

Few studies have evaluated the vasorelaxant effect of the genus *Ligusticum* (Umbelliferae). The ethanol extract of *L. wallichii* showed a maximal relaxant effect at 3000 μg/mL in isolated rat aorta [17]. The maximal relaxant effect of *L. sinensis* and *L. jeholense* essential oil was 76.7%–99.6% and 80.8% at 990 μg/mL, respectively [16]. In the study, the maximal relaxant effect of LJE was observed at 500 μg/mL. Considering the increasing interest in traditional medicines, *L. jeholense* may represent a potential candidate for the treatment of hypertension at lower doses than other genus *Ligusticum* therapeutics.

### 2.2. Effect of LJE on Endothelium-Intact or Endothelium-Denuded Aortic Rings Pre-Contracted with PE or KCl

We investigated the concentration-dependent vasorelaxant effect of LJE (30–500 μg/mL) on endothelium-intact and endothelium-denuded aortic rings pre-contracted with PE (1 μM) or KCl (60 mM) in standard KH buffer. The vasorelaxant effect of LJE was calculated as a percentage of the relaxation in response to PE and KCl on the aortic rings.

**Figure 2:**
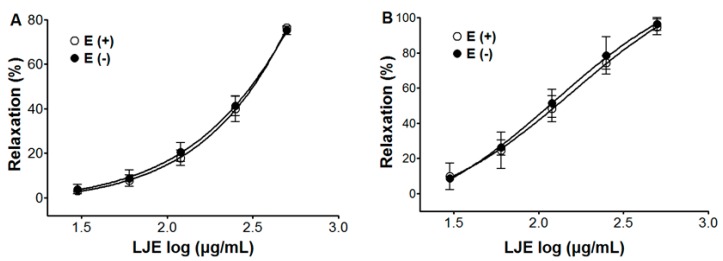
Concentration-dependent relaxant effect of LJE on PE (1 μM) (**A**) or KCl (60 mM); (**B**) pre-contracted aortic rings with [(E+)] or without [(E−)] endothelium. Values are expressed as mean ± SEM (*n* = 5–7).

LJE caused concentration-dependent relaxation in both endothelium-intact and endothelium-denuded aortic rings pre-contracted by PE or KCl treatment. The maximal relaxant effect on PE-induced contraction was 76.4 ± 1.2% and 75.2 ± 2.0% for endothelium-intact and endothelium-denuded aortic rings, respectively (Figure 2). And for KCl-induced contraction, the maximal relaxant effect was 94.6 ± 4.5% and 96.3 ± 3.6% for endothelium-intact and endothelium-denuded aortic rings, respectively (Figure 2).

The endothelial cell plays a major role in the vascular system, as it secretes regulated mediators or alters surface protein expressions that are vital for human homeostasis [18]. LJE caused concentration-dependent relaxation regardless of endothelial function. These results suggested that the vasorelaxant effect of LJE was endothelium independent.

### 2.3. Effect of LJE on Endothelium-Intact Aortic Rings Pre-Incubated with l-N~Nitro Arginine Methyl Ester (l-NAME), 1-H-[1,2,4]-Oxadiazolo-[4,3-α]-quinoxalin-1-one (ODQ), or Methylene Blue (MB)

We investigated the vasorelaxant effect of LJE (30–500 μg/mL) on the nitric oxide (NO) synthesis pathway in endothelium-intact aortic rings that were pre-incubated with l-NAME (10 μM) for 20 min before PE (1 μM) pre-contraction. We investigated the vasorelaxant effect of LJE on the NO-cyclic guanosine monophosphate (cGMP) pathway in endothelium-intact aortic rings that were pre-incubated with ODQ (10 μM) or MB (10 μM) for 20 min before PE pre-contraction. Compared to the control, the vasorelaxant effect of LJE was calculated as a percentage of the relaxation in response to l-NAME, ODQ, or MB pre-treatment on the aortic rings.

**Figure 3:**
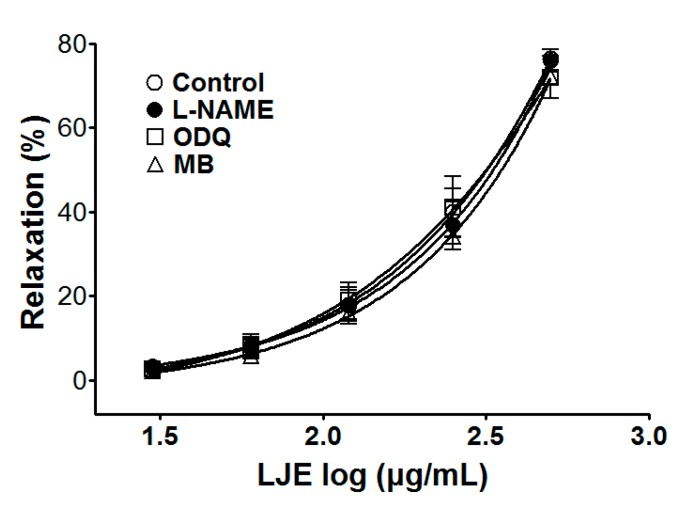
Concentration-dependent relaxant effect of LJE on PE (1 μM)-pre-contracted aortic rings in the presence or absence (control) of l-NAME (10 μM), ODQ (10 μM), or MB (10 μM). Values are expressed as mean ± SEM (*n* = 5–7).

Incubation with l-NAME, ODQ, or MB did not affect LJE-induced relaxation of endothelium-intact aortic rings pre-contracted by PE treatment. In the presence and absence of l-NAME, the maximal relaxant effect was 76.4 ± 1.2% and 76.0 ± 2.7%, respectively (Figure 3). The maximal relaxant effect in the presence and absence of ODQ was 72.0 ± 5.0% and 76.4 ± 1.2%, respectively (Figure 3). In incubation with MB, a maximal relaxant effect was of 73.1 ± 3.9% compared to control 76.4 ± 1.2% (Figure 3).

Endothelium induces not only vasoconstriction via the generation of endothelin, prostanoids, and conversion of angiotensin I to angiotensin II at the endothelial surface, but also vasorelaxation via the secretion of NO, prostacyclin, and endothelium-derived hyperpolarizing factor (EDHF). In endothelial cells, NO is generated from L-arginine by endothelial NO synthase (eNOS) activation, which is stimulated by the calcium-calmodulin complex. In the vascular smooth cells, NO gas activates soluble guanylate cyclase (sGC), which increases cyclic GMP (cGMP) and leads to cGMP-mediated vasodilation [19,20,21].

To investigate endothelium-derived vasorelaxation, the various inhibitors of LJE-induced vasorelaxation were used. Pre-treatment with l-NAME (NOS inhibitor), ODQ, or MB (sGC inhibitor) did not affect the vasorelaxant effects of LJE. These results suggested that the vasorelaxant effect of LJE did not have a relationship with the direct NO pathway, NO-cGMP pathway.

### 2.4. Effect of LJE on Endothelium-Intact Aortic Rings Pre-Incubated with Indomethacin

We examined the vasorelaxant effect of LJE (30–500 μg/mL) on the prostacyclin pathway in endothelium-intact aortic rings, that were pre-incubated with indomethacin (1 μM) for 20 min before PE (1 μM) pre-contraction. Compared to the control, the vasorelaxant effect of LJE was calculated as a percentage of the relaxation in response to indomethacin pre-treatment on the aortic rings.

**Figure 4:**
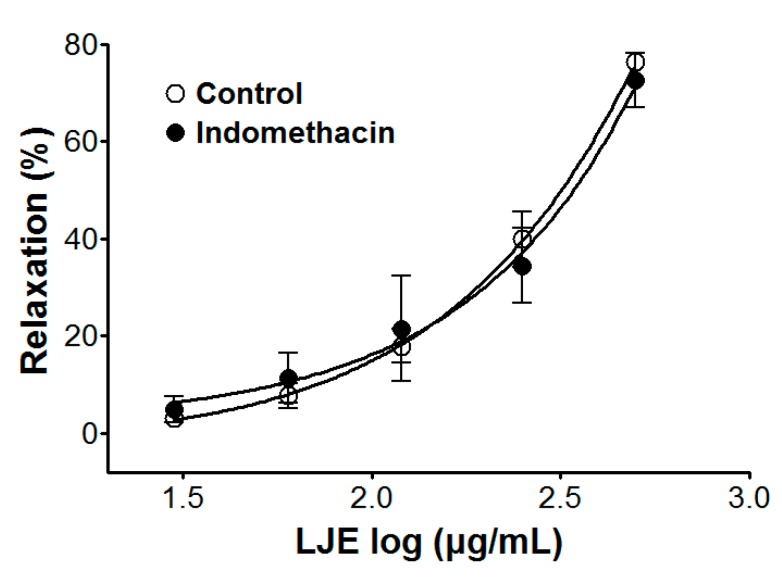
Cumulative concentration-response curves to LJE on PE (1 μM)-pre-contracted aortic rings in the presence or absence (control) of indomethacin (1 μM). Values are expressed as mean ± SEM (*n* = 4–7).

Incubation with indomethacin did not affect LJE-induced relaxation of endothelium-intact aortic rings pre-contracted by PE treatment. In the presence and absence of indomethacin, the maximal relaxant effect was 72.6 ± 5.5% and 76.4 ± 1.2%, respectively (Figure 4).

Prostacyclin, which is produced by cyclooxygenase-1 from arachidonic acid, increases 3′-5′-cyclic adenosine monophosphate (cAMP), which leads to vascular smooth muscle relaxation as a result [22]. Pre-treatment with indomethacin (a non-selective cyclooxygenase inhibitor) did not affect the vasorelaxant effects of LJE. This result suggested that the vasorelaxant effect of LJE did not have a relationship with vascular prostacyclin pathway.

### 2.5. Effect of LJE on Endothelium-Intact Aortic Rings Pre-Incubated with Atropine

We examined the vasorelaxant effect of LJE (30–500 μg/mL) from stimulation of muscarinic receptors in endothelium-intact aortic rings, that were pre-incubated with atropine (1 μM) for 20 min before PE (1 μM) pre-contraction. Compared to the control, the vasorelaxant effect of LJE was calculated as a percentage of the relaxation in response to atropine pre-treatment on the aortic rings.

**Figure 5:**
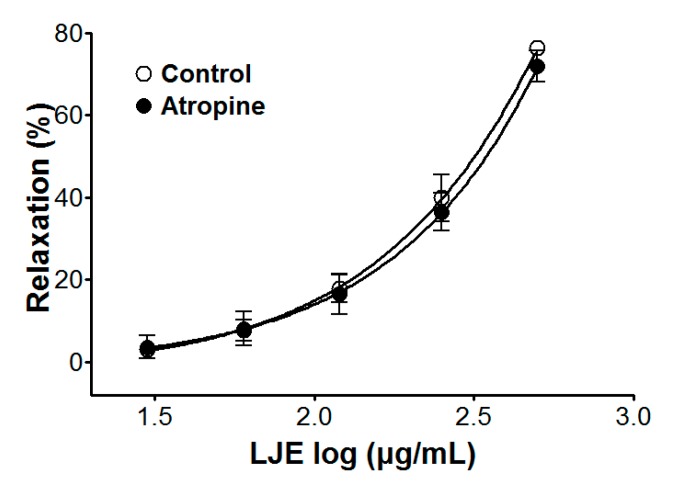
Cumulative concentration-response curves to LJE on PE (1 μM)-pre-contracted aortic rings in the presence or absence (control) of atropine (1 μM). Values are expressed as mean ± SEM (*n* = 5–7).

Incubation with atropine did not affect LJE-induced relaxation of endothelium-intact aortic rings pre-contracted by PE treatment. In the presence and absence of atropine, the maximal relaxant effect was 70.9 ± 3.8% and 76.4 ± 1.2%, respectively (Figure 5). Pre-treatment with atropine (a muscarinic receptor antagonist) did not affect the vasorelaxant effects of LJE. This result suggested that LJE was not associated with the muscarinic receptor.

### 2.6. Effect of LJE on Endothelium-Intact Aortic Rings Pre-Incubated with Various K^+^ Channel Blockers

We examined the vasorelaxant effect of LJE (30–500 μg/mL) in endothelium-intact aortic rings, that were pre-incubated with a K^+^ channel blocker such as tetraethylammonium (TEA, 5 mM), glibenclamide (10 μM), or 4-aminopyridine (4-AP, 1 mM) for 20 min before PE (1 μM) pre-contraction. Compared to the control, the vasorelaxant effect of LJE was calculated as a percentage of the relaxation in response to K^+^ channel blockers pre-treatment on the aortic rings.

**Figure 6:**
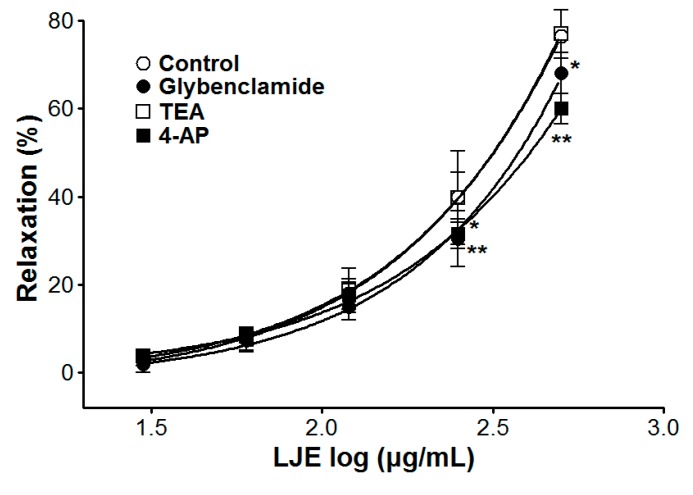
Cumulative concentration-response curves to LJE on endothelium-intact aortic rings pre-contracted with PE (1 μM) in the presence or absence (control) of glibenclamide (10 μM), TEA (5 mM), or 4-AP (1 mM). Values are expressed as mean ± SEM (*n* = 5–7). * *p* < 0.05, ** *p* < 0.01 *vs.* control.

The vasorelaxant effects of LJE on PE pre-contracted endothelium-intact aortic rings were altered by incubation with K^+^ channel blockers including glibenclamide or 4-AP. In the presence of glibenclamide or 4-AP, the LJE-induced maximal relaxant effect was of 68.1 ± 4.7% and 60.0 ± 3.4%, respectively (Figure 6). Incubation with TEA did not affect LJE-induced relaxation of endothelium-intact aortic rings pre-contracted by PE treatment (Figure 6).

In this study, removal of endothelial function or pre-treatment with l-NAME did not inhibit the relaxant effects of LJE. Thus, we concluded that the vasorelaxant effects of LJE might be due to the vascular smooth muscle.

The contraction and relaxation of vascular smooth muscle is regulated by the membrane potential through changes in K^+^ channel activity. The K^+^ channel activity is a main mechanism of vasoconstriction and vasodilation. In vascular smooth cells, the vascular activity is regulated by the membrane potential through changes in K^+^ channel activity, which changes activity of voltage-dependent Ca^2+^ channels [23]. To investigate the possibility that the vasorelaxant effects of LJE are mediated via K^+^ channels, various K^+^ channel blockers such as glibenclamide (K_ATP_ channels blocker, a highly selective blocker of ATP-sensitive K^+^ channels), TEA (K_Ca_ channels blocker, a blocker of big Ca^2+^-activated K^+^ channels), and 4-AP (K_V_ channels blocker, a predominant blocker of voltage-gated K^+^ channels) were used [24]. The vasorelaxant effect of LJE was partly attenuated by pre-treatment with glibenclamide or 4-AP as K^+^ channel blocker. We found that the vasorelaxant effects of LJE on the rat aortic rings are related to K^+^ channels such as K_ATP_ channels and K_V_ channels.

### 2.7. Effect of LJE on Extracellular Ca^2+^-Induced Contraction (via Receptor-Operative Ca^2+^ Channels or Voltage-Dependent Ca^2+^ Channels)

We examined the vasorelaxant effect of LJE (120–500 μg/mL) on extracellular Ca^2+^-induced contractions via receptor-operative Ca^2+^ channels (ROCCs) and voltage-dependent Ca^2+^ channels (VDCCs), by PE or KCl pre-treatment, respectively. We tested the contraction response induced by calcium chloride (CaCl_2_, 0.3–10 mM) in the endothelium-denuded aortic rings by PE (1 μM) or KCl (60 mM) pre-contraction in Ca^2+^-free KH buffer with and without (control) LJE preincubation for 10 min. Compared to the control, the contraction responses induced by CaCl_2_ were calculated as a percentage in the presence and absence (control) of LJE pre-treatment.

**Figure 7:**
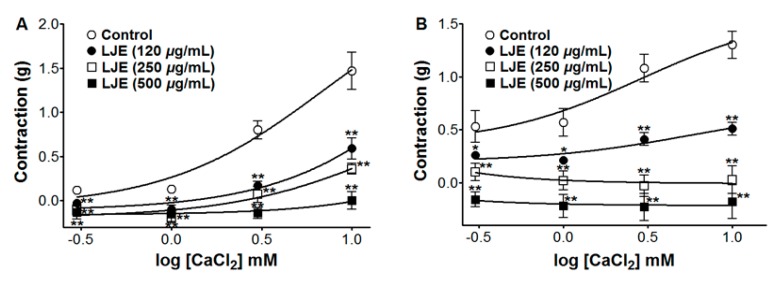
Inhibitory effect of LJE (120–500 μg/mL) on the contraction induced by extracellular Ca^2+^ in endothelium-denuded rat thoracic aorta rings that were pre-contracted with PE (1 μM) (**A**) or KCl (60 mM) (**B**) in the presence or absence (control) of LJE. Values are expressed as mean ± SEM (*n* = 4). * *p* < 0.05, ** *p* < 0.01 *vs.* control.

In Ca^2+^-free KH buffer, the cumulative addition of CaCl_2_ (0.3–10 mM) induced progressively increased tension in the rat thoracic aorta rings. As shown in Figure 7, LJE (120–500 μg/mL) pre-incubation significantly inhibited the contractions induced by extracellular CaCl_2_ (10 mM) and the contraction at LJE (500 μg/mL) concentration was decreased to 0.00 ± 0.10 g and −0.18 ± 0.16 g *vs.* the control group 1.47 ± 0.21 g and 1.30 ± 0.13 g, in cells pre-contracted by PE and KCl, respectively (Figure 7).

The contraction and relaxation of vascular smooth muscle is regulated by Ca^2+^ entry from the extracellular space through ROCCs or VDCCs in the plasma membrane, through Ca^2+^ release from intracellular Ca^2+^ stores (sarcoplasmic reticulum), protein kinase C (PKC) activation, and a Ca^2+^ sensitization mechanism [25]. PE induced the influx of extracellular Ca^2+^ by activating ROCCs and KCl induced Ca^2+^ influx through VDCCs [26]. LJE inhibited vasoconstriction induced by Ca^2+^ supplementation in the aortic rings that were pre-contracted with PE or KCl in Ca^2+^-free KH buffer. These results suggested that LJE significantly inhibited the entry of extracellular Ca^2+^ via ROCCs or VDCCs activated by PE or KCl.

### 2.8. Effect of LJE and SK & F96365 on PE-Induced Contraction in the Presence of Nifedipine

The effect of LJE on Ca^2+^ influx through ROCCs was investigated by determining the effect of LJE (500 μg/mL) and the ROCCs blocker SK & F 96365 (50 μM) on PE (1 μM)-induced contraction in the presence of the VDCCs blocker nifedipine (10 μM). PE was applied twice in the presence of nifedipine; the aortic rings were treated with LJE or SK & F 96365 before the second application of PE.

**Figure 8:**
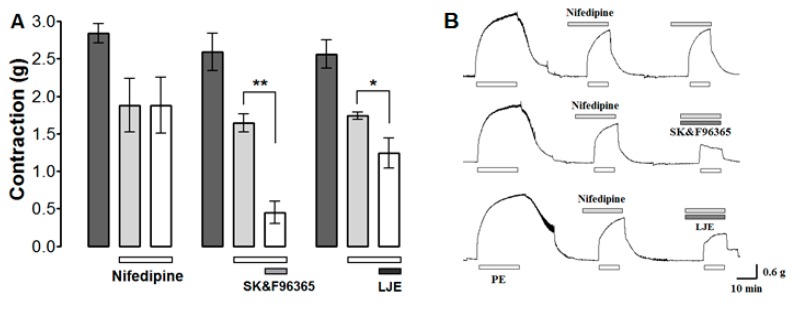
The effects (**A**) and traces (**B**) of LJE (500 μg/mL) and SK & F96365 (50 μM) in the presence of nifedipine (10 μM) on PE-induced contraction. Values are expressed as mean ± SEM (*n* = 4). * *p* < 0.05, ** *p* < 0.01.

Nifedipine inhibited the contraction induced by PE. Further inhibition was observed after the application of LJE (500 μg/mL) or SK & F 96365 (Figure 8). Nifedipine is an inhibitor of voltage-gated Ca^2+^ entry and SK & F 96365 is a selective inhibitor of receptor-mediated Ca^2+^ entry [27]. A combination of SK & F 96365 and nifedipine decreased further PE-induced contractions. As a result, nifedipine blocked VDCCs at first and SK & F 96365 blocked ROCCs in sequence. Likewise, LJE (500 μg/mL) decreased PE-induced contractions in the presence of nifedipine, suggesting that LJE inhibits the entry of extracellular Ca^2+^ via ROCCs activated by PE.

Many compounds, including linoleic acid, ferulic acid, and beta-sitosterol, were isolated from *L. jeholense* root and rhizoma [15]. The main components of essential oil from this plant were reported to be *m*-diaminobenzene (68.2%), ligustilide (10.1%), *p*-vinylguaiacol (3.5%), apiol (2.0%) [7], or β-phellandrene (33.3%) [5]. The vasorelaxant effects of these few compounds have been reported previously. Ferulic acid inhibits angiotensin II-induced constriction of the vascular smooth muscle cells by regulating cell cycle progression [28]. Linoleic acid brings about vasorelaxation and hyperpolarization by activating Na^+^/K^+^-ATPase pumps [29]. Our results also showed that LJE-induced vasorelaxation was related to ATP-sensitive and voltage-sensitive K^+^ channels (K_ATP_ and K_V_ channels). It was found in previous studies that the vasorelaxant effects of ligustilide were not altered by endothelium removal or inhibition of adenylate cyclase, sGC [8]. Likewise, in our study, it was found that the vasorelaxant effects of LJE were endothelium-independent and not related to the NO-cGMP pathway. Hence, it is likely that linoleic acid, ferulic acid, or ligustilide could be responsible for the vasorelaxant effects of this plant. *L. jeholense* consists of various compounds, and further investigation is required to examine the mechanism of active compounds of this plant in rat aortic rings.

## 3. Experimental Section

### 3.1. Chemicals and Drugs

Modified KH buffer powder, PE, KCl, l-NAME, ODQ, MB, indomethacin, atropine, TEA, glibenclamide, 4-AP, CaCl_2_, ethylene glycol bis (2-aminoethylether)-*N*,*N*,*N′*,*N′*-tetraacetic acid (EGTA), SK & F96365, nifedipine, and dimethyl sulfoxide (DMSO) were purchased from Sigma Aldrich (St. Louis, MO, USA). All other reagents were of analytical purity.

### 3.2. Plant Material and Extraction

*L. jeholense* root and rhizoma, were collected between 37°13′N to 37°21′N latitude and 128°43′E to 128°55′E longitude in Jeongseon, Gangwon Province, Republic of Korea, in August 2014. Professor Hocheol Kim of Kyung Hee University identified the plant. A voucher specimen of the plant (VS14080201) was deposited in the herbarium of the College of Korean Medicine, Kyung Hee University, Seoul, Republic of Korea. Dried *L. jeholense* root and rhizoma (100.0 g) was extracted three times with 1 L 100% MeOH for 3 h in a reflux apparatus at 70 ± 5 °C. After filtration, the extract was evaporated in a rotary vacuum evaporator (N-N series, EYELA, Tokyo, Japan) at 60 °C and lyophilized in a freeze-dryer (Operon™, Seoul, Korea) to obtain a dark brown powder (20.9 g) of crude extract. LJE powder was accurately weighed (0.1 g), suspended in 1 mL KH buffer, and placed into an ultrasonic device for 1 min for solubilization. The powder was completely dissolved, and the color was light brown.

### 3.3. Animals

We used male Sprague-Dawley rats (weight, 240–260 g; Raonbio, Yongin, Gyeonggi` Province, Korea). All animal procedures were conducted according to the animal welfare guidelines and were approved [KHUASP(SE)-15-013] by the Kyung Hee University Institutional Animal Care and Use Committee. All rats were acclimated under standard laboratory conditions (22 ± 2 °C; lighting, 07:00–19:00) and food and water were given *ad libitum*.

### 3.4. Preparation of Rat Aortic Rings

Rats were anesthetized by exposure to ether, the thoracic aorta was removed and immersed in KH buffer [composition (mM): NaCl, 118.0; KCl, 4.7; MgSO_4_, 1.2; KH_2_PO_4_, 1.2; CaCl_2_, 2.5; NaHCO_3_, 25.0; and glucose, 11.1; pH 7.4], and then aerated with a gas mixture of 95% O_2_–5% CO_2_ at 37 °C. After carefully removing the connective tissue and fat surrounding the aorta, the aorta was cut into 2-mm-long rings and suspended in organ chambers containing 10 mL KH buffer at 37 °C. The rings were suspended between two tungsten stirrups and one stirrup was connected to an isometric force transducer (Grass Instrument Co., West Warwick, RI, USA). The aortic ring segments were incubated under no tension for 30 min and left to equilibrate for 1 h at an optimal resting tension of 1.2 g. The KH buffer was refreshed every 15–20 min during the equilibration period for 90 min. The changes in tension of the aortic rings were recorded using isometric transducers connected to a data acquisition system (PowerLab, ADI instrument Co., Bella Vista, New South Wales, Australia). When necessary, the endothelium layer was removed by gently rubbing inside the lumen using a thin polyethylene stick. The presence of functional endothelium was checked by the ability of ACh (10 μM) to induce more than 80% relaxation in PE (1 μM)-contracted aorta rings. Endothelium-denudation was considered effectively removed when ACh caused less than 10% relaxation. Ca^2+^-free KH buffer was prepared by removing CaCl_2_ and adding EGTA (1 mM). The antagonist and inhibitor concentrations were selected in the same manner as described previously [30,31].

### 3.5. Data Analysis

Results are expressed as mean ± standard error of mean (SEM). Statistical comparisons were made using Student’s *t*-test. All statistical analyses were performed by using SPSS (version 21.0) statistical analysis software (SPSS Inc., Chicago, IL, USA). *P* values less than 0.05 were considered statistically significant.

## 4. Conclusions

In conclusion, (1) the vasorelaxant effects of LJE were not related to the direct NO pathway, NO-cGMP pathway, vascular prostacyclin (PGI_2_) pathway, or muscarinic receptors transduction pathway; (2) K^+^ channels were partly related to LJE-induced vasorelaxation; and (3) LJE relaxed the aortic rings by blocking the entry of extracellular Ca^2+^ via ROCCs and VDCCs.
